# Skeletal Muscle Regeneration by the Exosomes of Adipose Tissue-Derived Mesenchymal Stem Cells

**DOI:** 10.3390/cimb43030104

**Published:** 2021-10-09

**Authors:** Seong-Eun Byun, Changgon Sim, Yoonhui Chung, Hyung Kyung Kim, Sungmoon Park, Do Kyung Kim, Seongmin Cho, Soonchul Lee

**Affiliations:** 1 Department of Orthopaedic Surgery, CHA Bundang Medical Center, CHA University School of Medicine, Gyeonggi-do 13488, Korea; sonofos@daum.net (S.-E.B.); jeongyunhui92@gmail.com (Y.C.); tjdans4638@naver.com (S.P.); fkzhfjqm@naver.com (D.K.K.); a186055@chamc.co.kr (S.C.); 2 CHA Graduate School of Medicine, 120 Hyeryong-ro, Pocheon 11160, Korea; simchanggon@naver.com; 3 Department of Pathology, Seoul National University Bundang Hospital, 82 Gumi-ro 173-beon-gil, Bundang-gu, Seongnam 13620, Korea; hyungkyungjkim@gmail.com

**Keywords:** mesenchymal stem cell, exosome, muscle, satellite cell, regeneration

## Abstract

Profound skeletal muscle loss can lead to severe disability and cosmetic deformities. Mesenchymal stem cell (MSC)-derived exosomes have shown potential as an effective therapeutic tool for tissue regeneration. This study aimed to determine the regenerative capacity of MSC-derived exosomes for skeletal muscle regeneration. Exosomes were isolated from human adipose tissue-derived MSCs (AD-MSCs). The effects of MSC-derived exosomes on satellite cells were investigated using cell viability, relevant genes, and protein analyses. Moreover, NOD-SCID mice were used and randomly assigned to the healthy control (*n* = 4), muscle defect (*n* = 6), and muscle defect + exosome (*n* = 6) groups. Muscle defects were created using a biopsy punch on the quadriceps of the hind limb. Four weeks after the surgery, the quadriceps muscles were harvested, weighed, and histologically analyzed. MSC-derived exosome treatment increased the proliferation and expression of myocyte-related genes, and immunofluorescence analysis for myogenin revealed a similar trend. Histologically, MSC-derived exosome-treated mice showed relatively preserved shapes and sizes of the muscle bundles. Immunohistochemical staining revealed greater expression of myogenin and myoblast determination protein 1 in the MSC-derived exosome-treated group. These results indicate that exosomes extracted from AD-MSCs have the therapeutic potential for skeletal muscle regeneration.

## 1. Introduction

The skeletal muscle, accounting for 30–40% of the total body weight [1], is important not only as a facilitator of physical activity by generating force but also as a paracrine or endocrine organ affecting the bones and the body’s metabolism [2]. Hence, muscle defects and injuries due to trauma or muscle loss caused by aging or chronic/genetic diseases can negatively affect various aspects and measures of health. Several pharmacologic agents, including myostatin inhibitors and androgenic hormone derivatives, have been used in attempts to regenerate the skeletal muscle [3,4,5]. Despite the promising results of a few trials [6,7], the lack of functional improvement and unexpected complications has prevented the clinical use of these drugs in muscle regeneration [3,4,5]. Thus, cell-based regenerative approaches are urgently needed for therapy of skeletal muscle damage.

If the muscle is severely and extensively injured, the number of local endogenous cells is often not adequate to restore tissue continuity or function in the defect [8]. Mesenchymal stem cells (MSCs) are an attractive alternative treatment modality in tissue regeneration because they can supplement the shortage of cells. MSCs can also differentiate into many cell types, including osteoblasts, chondrocytes, adipocytes, and myoblasts [9].

Among various sources of MSCs, the adipose tissue provides numerous benefits including easy access, availability, and more excellent stem-cell proliferation rates compared with the bone marrow or birth-associated tissues [10,11,12]. Adipose tissue-derived MSCs (AD-MSCs) have shown their multipotent feature to differentiate into traditional mesenchymal lineages including osteogenic, myogenic, chondrogenic, and adipogenic [10,11,13]. Additionally, multiple angiogenic and antiapoptotic cytokines, including growth factors, such as insulin-like growth factor-1 (IGF-1), vascular endothelial growth factor (VEGF), and hepatocyte growth factor (HGF), were shown to be secreted by AD-MSCs, promoting tissue regeneration and reducing tissue damage [14,15,16]. Although the precise therapeutic mechanisms have not been clearly elucidated, this paracrine-mediated effect of AD-MSCs has been suggested as an important mechanism of muscle regeneration without direct cell replacement [17]. However, several drawbacks of transplantation of AD-MSCs including poor engraftment efficiency, unregulated differentiation, the possibility for tumor development, and undesirable immunological responses have been reported [9,18,19,20].

In this context, the feasibility of exosomes derived from AD-MSCs has recently been introduced [21]. Exosomes that contain proteins and RNAs, including messenger RNA and microRNA, can transfer signal molecules, thereby facilitating the regeneration of muscle tissue. Several studies have revealed the potential of exosomes secreted by AD-MSCs in regenerative medicine [22,23].

Therefore, in this study, the muscle-regenerating effect of exosomes derived from human AD-MSCs was evaluated using muscle defect models in rodents. We hypothesized that the exosomes from human AD-MSCs exert a novel paracrine effect on skeletal muscle repair in the area of a muscle defect in addition to their secretion of cytokines, chemokines, or growth factors.

## 2. Materials and Methods

### 2.1. Cell Culture

Human AD-MSCs were purchased from PromoCell (Heidelberg, Germany) and were cultured in MSC growth medium 2 (PromoCell, c-12977) supplemented with 10% fetal bovine serum (System Bioscience, Palo Alto, CA, USA) and 1% penicillin–streptomycin (HyClone, Logan, UT, USA). AD-MSCs from the second to fourth passages were used for experiments. Human skeletal muscle satellite cells (HSkMSCs) were obtained from ScienCell (San Diego, CA, USA) and were grown in HSkMSC growth medium (ScienCell) supplemented with growth factors, penicillin, and streptomycin. For the growth and differentiation of HSkMSCs, the cells were cultured in skeletal muscle cell growth medium until they reached approximately 60%–80% confluence, and then the medium was replaced with skeletal muscle cell differentiation medium supplemented with DMEM, 2% horse serum (HyClone), and 1% penicillin–streptomycin. The cells were cultured for 9 days, and the media were replaced every 2–3 days.

When the AD-MSCs reached 80% confluence, the medium was replaced by fresh medium supplemented with 10% exosome-depleted FBS and 1% penicillin–streptomycin. The cell-cultured conditioned medium was collected after incubation for 48 h.

### 2.2. Isolation and Analysis of Exosomes

MSC-derived exosomes were isolated using ExoQuick-TC (System Bioscience, Palo Alto, CA, USA) according to the manufacturer’s protocol. The exosome pellets were resuspended in phosphate-buffered saline (PBS). Exosome concentration was assessed using a Pierce™ BCA Protein Assay Kit (Thermo Fisher Scientific, Indianapolis, IN, USA). The protein levels of CD9, CD63, and CD81, the representative markers of exosomes, were detected using Western blot. Transmission electron microscopy (TEM, JEM-1011,Tokyo, Japan) was used to observe the morphology and distribution of the exosomes. The exosomes were fixed using 4% paraformaldehyde for 30 min. The mixture was embedded on carbon-coated copper grids and was dried for 10 min at room temperature. The dried mixture was stained with 1% uranyl acetate for 5 min. TEM images were then obtained using TEM at 80 kV. The resuspended exosome pellets were diluted to 100 μg/mL using PBS and loaded into the cuvette. Then, the size distribution of exosomes was measured using a particle size and zeta potential analyzer (DLS, ELS-1000ZS, Osaka, Japan).

### 2.3. Western Blotting

The exosomes were lysed in PRO-PREP (Intron Biotechnology, Seoul, South Korea) according to the manufacturer’s protocol. The protein was electrophoresed on 12% SDS-PAGE gel and was transferred to a PVDF membrane (Millipore, Billerica, MA, USA). The membrane was blocked with 5% skim milk in T-TBS (10 mM Tris, 150 mM NaCl, and 0.1% Tween 20) for 1 h at room temperature and was subsequently incubated with anti-CD9, anti-CD63, or anti-CD81 (Invitrogen, Carlsbad, CA, USA) (see Table 1 for details on antibodies) overnight at 4 °C. After vigorous washing in T-TBS, the blots were incubated with horseradish peroxidase (HRP)-tagged anti-rabbit or anti-mouse secondary antibodies from Santa Cruz Biotechnology (Dallas, TX, USA) for 1 h. The labeled proteins were visualized using the ChemiDoc™ XRS imaging system (Bio-Rad Laboratories, Inc., Hercules, CA, USA) using an enhanced chemiluminescence kit (GE Healthcare Life Sciences, Pittsburgh, PA, USA). All chemical reagents were purchased from Bio-Rad Laboratories (Hercules, CA, USA).

### 2.4. Quantitative Real-Time Polymerase Chain Reaction (qRT-PCR)

Total RNAs were isolated from satellite cells with or without exosome treatment using a TRIzol reagent, and reverse transcriptions were conducted using an iScript™ cDNA Synthesis Kit (Bio-Rad Lab., Hercules, CA, USA) as per the manufacturer’s instructions. To quantify the transcripts of the interest genes (Table 2), qRT-PCR was conducted using an SYBR™ Select Master Mix (Applied Biosystems, Austin, TX, USA) on a Bio-Rad CFX96 Real-Time PCR Detection System. The transcript levels of the genes of interest were normalized to GAPDH and were calculated using the 2^−ΔΔCt^ method.

### 2.5. Cell Proliferation Assay

Cell proliferation was detected using a CCK-8 solution (Dojindo, Kumamoto, Japan) as per the manufacturer’s instructions. Briefly, HSkMSCs were seeded into 96-well plates at 1.5 × 10^3^ cells/well and were incubated at 37 °C in 5% CO_2_ for 24 h. Then, the culture medium was replaced with 100 μL of fresh medium containing 0, 12.5, and 25 μg/mL of exosomes. The medium was replaced every 3 days. After incubation for 3 or 9 days, 10 μL of CCK-8 was added, plates were incubated at 37 °C for 2 h, and the absorbance was measured at 450 nm.

### 2.6. Immunofluorescent Staining

The HSkMSCs were seeded at 2 × 10^4^ cells/well into a 24-well plate on coverslips and were grown in a growth medium for 24 h; then, the culture medium was replaced with a fresh medium containing 0 and 25 μg/mL of exosomes. After 9 days, the cells were washed twice using cold PBS, fixed with 4% paraformaldehyde/PBS for 1 h at 4 °C, washed three times with PBS, and permeabilized using 0.3% Triton X-100 for 20 min at room temperature. Later, cells were blocked with 3% BSA/PBS for 1 h. The cells were incubated overnight at 4 °C with anti-myogenin (MYOG) (sc-12732, Santa Cruz, CA, USA) or anti-myod (MYOD) (sc-377460, Santa Cruz, USA) and were then incubated with anti-mouse IgG–FITC secondary antibody (Santa Cruz). The cells were counterstained using 1 μg/mL 4,6-diamidino-2-phenylindole (DAPI) (Sigma-Aldrich) for 1 min and were then observed with a Zeiss LSM 510 laser scanning confocal microscope (Carl Zeiss Microimaging, Thornwood, NY, USA).

### 2.7. Animals

Six week old NOD-SCID mice were purchased from Orient Bio (Seongnam, Korea) and were maintained at the barrier facility of the Laboratory Animal Research Center of CHA University. The mice were group-housed in ventilated cages, were given acidified water and an irradiated rodent diet, and were maintained on an automatic 12 h light and dark cycle. All experimental studies were performed according to and approved by the Institutional Animal Care and Use Committee of CHA University. The mice were divided into three experimental groups: healthy control (*n* = 4), gel alone (*n* = 6), and gel + exosome (*n* = 6) (Table 3).

### 2.8. Muscle Defect Surgery

**Figure 1:**
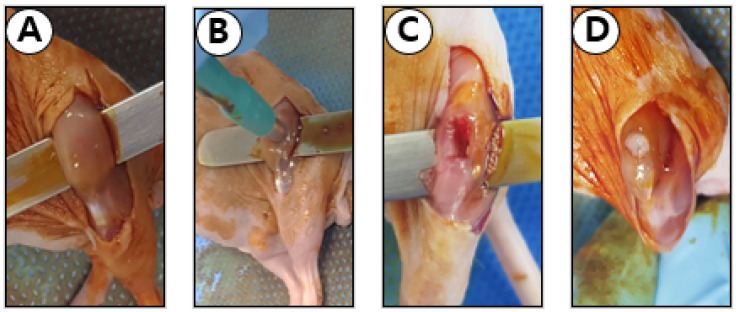
Surgical photo of the mouse muscle defect model. (**A**,**B**) After exposing the left quadriceps muscle of the mouse, a 2 mm^3^ muscle defect was created using a commercial skin biopsy punch. (**C**) The defected muscle is shown by elevating the leg using a tongue depressor. (**D**) The scaffold (fibrin gel) was placed, and the wound was closed layer by layer.

A muscle defect was created for the gel alone and gel + exosome groups (Figure 1). After the induction of general anesthesia, the hind limb was sterilized using povidone, followed by 70% ethanol. A 2 cm longitudinal incision was made over both hind limb muscles to expose the whole quadriceps muscle. The muscle defect was created by removing a 2 mm^3^ sized muscle using a commercial skin biopsy punch. If needed, hemostasis was performed by gently pressing the wound.

For the in situ casting of the exosome with fibrin gel, freshly harvested exosomes (2 mg/mL) were resuspended in 20 μL of fibrinogen solution, and the muscle defect was filled with the solution. Casting was induced by adding a thrombin solution (5 IU/2 μL). After a 2 min waiting period to ensure gel solidification, the wound was carefully covered with the fascia and skin and was secured using sutures. The fascia and skin were then sutured using a 4–0 polydioxanone suture (Ethicon, Cincinnati, OH, USA). For the gel alone group, the defect was cast with the fibrin gel alone without the incorporation of exosomes. For the healthy control group, mock surgery was performed. The skin and fascia on the hind limb were incised, and the muscle was exposed. Without additional intervention, the fascia and skin were closed. After 4 weeks of surgery, the mice were sacrificed. The quadriceps muscles were then carefully isolated and were weighed using an electronic balance.

### 2.9. Histologic Analyses

To histologically evaluate the muscle regeneration capacity of MSC-derived exosomes, the harvested tissues were fixed using 10% paraformaldehyde. After fixation, the tissue samples were dehydrated using a graded series of ethanol (70%, 95%, and 100%) using an automatic tissue processor unit (Leica, ASP 300S, Wetzlar, Germany). The dehydrated samples were soaked in xylene for transparent progress and were embedded into paraffin wax. The paraffin blocks were sectioned into 8–12 μm slices and deparaffinized with HistoClear. After deparaffinization, the antigen retrieval process was conducted. The slides were boiled in 0.01 M sodium citrate buffer for 15 min. After boiling, the slides were maintained at room temperature in the buffer for 20 min. The samples were then stained with hematoxylin and eosin.

To perform immunohistochemistry staining, the deparaffinized samples were stained with either MYOG (1:300, sc-12732, Santa Cruz, USA) or MYOD (1:300, sc-377460, Santa Cruz, USA) (see Table 1 for details on antibodies). For nonspecific protein blocking, 5% normal goat serum was used for 1 h at room temperature. The blocked samples were incubated with diluted primary antibodies for 1 h at room temperature. After primary antibody attachment processing, the samples were rinsed twice with TBST for 5 min. The secondary antibodies of goat anti-mouse IgG–HRP (1:200, ab6789, Abcam, Cambridge, MA, USA) and goat anti-rabbit IgG–HRP (1:200, ab6721, Abcam, Cambridge, MA, USA) were treated for 1 h at room temperature. After secondary antibody treatment, streptavidin peroxidase was added to the sectioned samples for 30 min in a dark room at room temperature. Peroxidase activity was developed using the AEC Substrate Chromogen (Dako, Carpinteria, CA, USA) and was rinsed using PBS. After rinsing, all tissue samples were mounted using a mounting medium and were covered using coverslips. The stained tissue images were obtained using a digital pathology scanner (Pannoramic 250 FLASH III, 3D Histech, Bubapest, Hungary).

### 2.10. Statistical Analyses

All data are presented as the mean ± standard deviation (SD). All statistical analyses were conducted using GraphPad PRISM software version 6 (GraphPad Software, Inc., San Diego, CA, USA) and software R (version 3.2.4; R Foundation for Statistical Computing). Data were tested for normality using the Kolmogorov–Smirnov and Shapiro–Wilk tests. When the data were normally distributed, the analysis of variance test (with post hoc tests of Bonferroni) was conducted, whereas the Kruskal–Wallis test was conducted to test the significance of data not normally distributed. Additionally, the Mann–Whitney U test was used to test the significance when only two groups were tested. Statistical significance was determined at the *p* < 0.05 level (* *p* < 0.05, ** *p* < 0.01, *** *p* < 0.001, and **** *p* < 0.0001). All in vitro experiments were conducted at least three times.

## 3. Results

### 3.1. Characterization of MSC-Derived Exosomes

**Figure 2:**
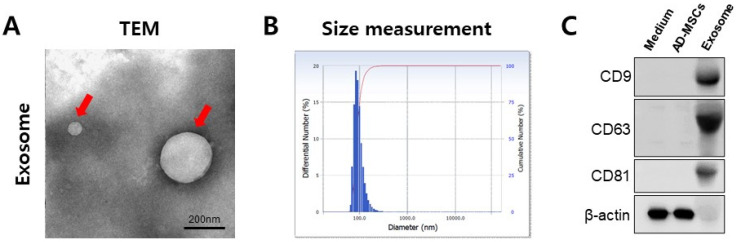
Characterization and quantification of exosome levels in media from human AD-MSCs. (**A**) Representative TEM image of exosomes. Red arrows indicate an exosome. Scale bar = 200 nm (**B**) Dynamic light scattering (DLS) analysis of exosomes. DLS result for the size distribution of the exosome is shown. (**C**) Western blot of CD9, CD63, and CD81 in exosomes, AD-MSCs, and medium.

Discrete vesicles of various sizes and diameters were observed using a TEM (Figure 2A). To determine the size distribution of the exosomes, the exosomes were measured by DLS. The DLS analysis of exosomes revealed an average diameter of 118 nm (Figure 2B). The Western blot of equivalent quantities of proteins showed an enrichment of exosome markers including CD9, CD81, and CD63 in the exosome samples compared with the medium and human AD-MSCs (Figure 2C). These results, along with the electron micrographs, indicate that the pellets isolated from the conditioned media of human AD-MSC culture by sequential centrifugation constituted a distinct set of secretory vesicles.

### 3.2. Effect of Exosomes on Satellite Cells

**Figure 3:**
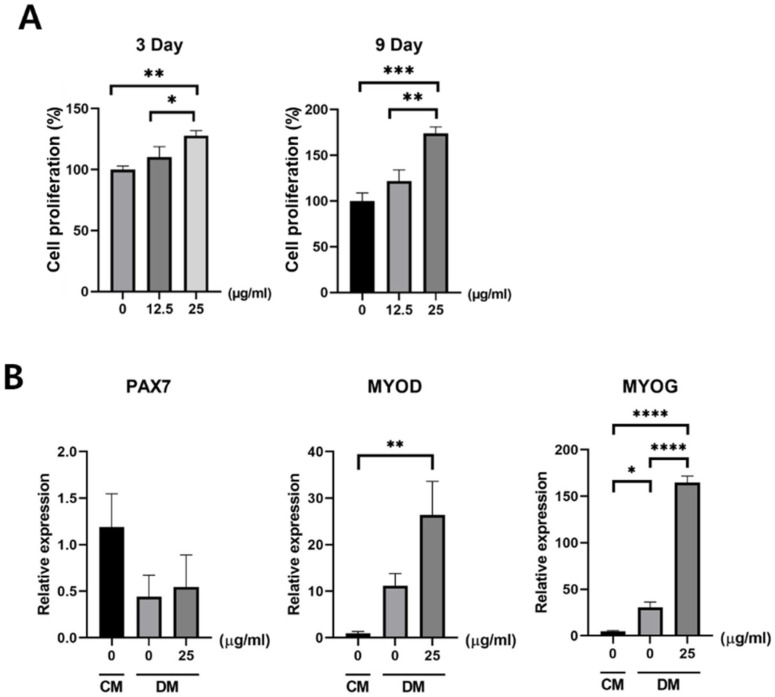
Changes in satellite cell proliferation and differentiation markers after exosome treatment. (**A**) Exosome treatment for each satellite cell culture (0, 12.5, and 25 μg/mL). Cell proliferation was measured using the CCK-8 solution method at 3 and 9 days after treatment. The satellite cells treated using 25 μg/mL of exosomes showed a significantly higher proliferation than that of the control both 3 and 9 days after treatment. (**B**) The qRT-PCR showed that MYOD was significantly increased by 25 μg/mL of exosome treatment compared with those without exosome treatment in differentiation media (DM). MYOG also increased by almost 41-fold, which implies that satellite cells successfully differentiated into myocytes. The *PAX7* gene decreased in DM compared with the control media. However, no statistical significance was observed; data are presented as the mean ± SD. * *p* < 0.05, ** *p* < 0.01, *** *p* < 0.001, and **** *p* < 0.0001 (*n* = 3).

**Figure 4:**
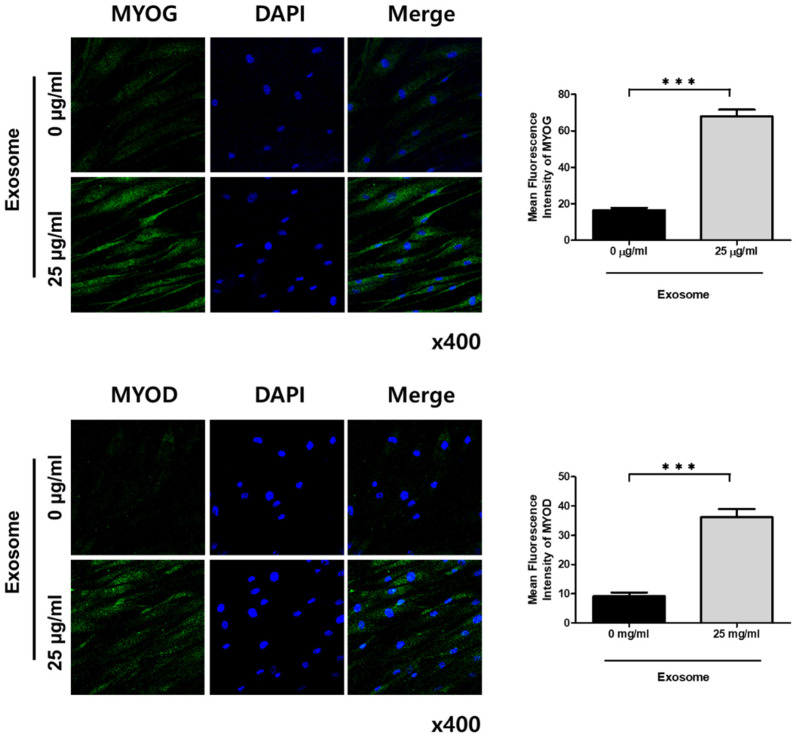
Changes in MYOG and MYOD protein expressions in the differentiation media of satellite cells were analyzed using immunofluorescence. To detect MYOG and MYOD protein expressions, treatment with anti-myogenin as the first antibody and anti-mouse IgG–FITC as the secondary antibody (green), followed by counterstaining using Dn knm API (blue), was conducted. The satellite cell group treated with exosomes showed a strong expression level throughout the cytoplasm, whereas the nontreated group showed a weak expression level. Relative mean fluorescence intensity was calculated using Image J for MYOG and MYOD. Data are presented as the mean ± SD. *** *p* < 0.001, (*n* = 3). The images were taken at 400× magnification.

An improved proliferation of the satellite cells was observed after exosome treatment and was found to be dose-dependent. The CCK-8 test revealed that cell proliferation was significantly enhanced with 25 µg/mL of exosome treatment compared with that of the control and cells treated with 12.5 µg/mL of exosomes after 3 days of treatment. The proliferation of exosome-treated satellite cells was further improved after 9 days of treatment. After 9 days of treatment, a significant difference in cell proliferation was found in the satellite cells treated with 25 µg/mL of exosomes compared with the control and cells treated with 12.5 µg/mL of exosomes (Figure 3A). PCR revealed that myogenic differentiation markers including MYOG and myoblast determination protein 1 (MYOD) were significantly increased. Although the satellite cell marker *PAX7* was decreased, this was not statistically significant, which means that the satellite cells were induced into myogenic differentiation by 25 µg/mL of exosome treatment (Figure 3B). Immunofluorescent staining also showed an enhanced expression of MYOG and MYOD proteins with 25 µg/mL of exosome treatment compared with that of the control (Figure 4).

### 3.3. Muscle Regeneration after Exosome Treatment

**Figure 5:**
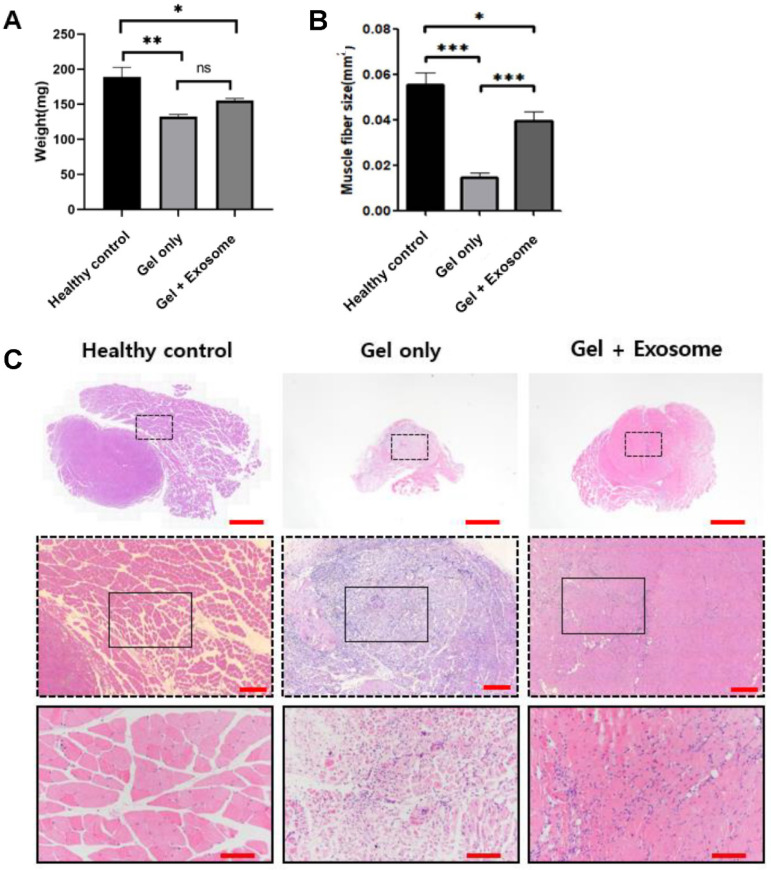
Gross muscle weight and histological results. (**A**) Four weeks after surgery, the quadriceps muscles were harvested and weighed. The gel + exosome group had a higher muscle weight than the gel-only group, but this was not statistically significant. (**B**) The muscle fiber size was greater in the gel + exosome group than in the gel-only group. (**C**) In the hematoxylin and eosin staining of the harvested quadriceps muscle of the defect site, the healthy control group showed a normal muscle fiber structure with different directions, as was expected, and the gel-only group had profound infiltration of inflammatory cells and collapse of muscle structure and integrity by muscle atrophy. However, in the gel + exosome group, the integrity of the muscle was re-established with an abundance of extracellular matrix. Moreover, the proportion of inflammatory cells was significantly reduced. Scale bar for top column, middle column, and bottom column = 1000, 200, and 100 µm, respectively. Data are presented as the mean ± SD. * *p* < 0.05, ** *p* < 0.01, *** *p* < 0.001, and ns: nonsignificant (*n* = 4).

Muscle regeneration was observed through gross inspection after harvesting the muscles. The weight of the injured muscles after sacrificing the mice showed statistically significant differences between groups. The muscles of the animals treated with 2 mg/mL of exosomes showed significantly higher weights than those of the negative control group (animals treated with gel only) (Figure 5A).

**Figure 6:**
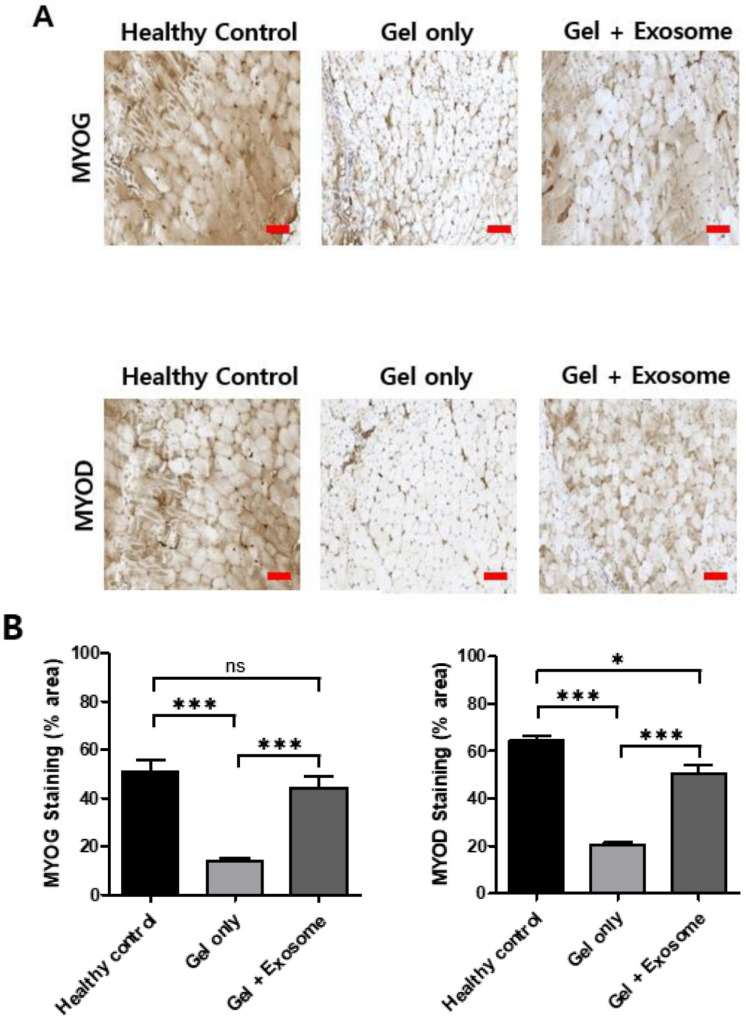
Immunohistochemistry of MYOG and MYOD proteins. (**A**) Increased expression of the MYOG and MYOD proteins was also observed in the exosome-treated group (gel + exosome) compared with the control (gel only) group in immunohistochemical staining, which indicated that the muscles regenerated. Scare bar = 100 μm. (**B**) Quantification of MYOD and MYOG staining was calculated using Image J. Data are presented as the mean ± SD. * *p* < 0.05, *** *p* < 0.001, and ns: nonsignificant (*n* = 4).

The negative control group showed necrosis and atrophy of the muscle fibers with morphological heterogeneity. Inflammatory cell infiltration was also observed during muscle repair. The exosome-treated group showed relatively preserved shapes and sizes of the muscles bundled with normal muscle regeneration. The muscle fiber size was greater in the exosome-treated group than in the negative control (Figure 5B,C). Immunohistochemical staining showed a greater expression of the MYOG and MYOD proteins in the exosome-treated group than in the negative control, indicating that the muscle’s ability to contract gradually recovered (Figure 6).

## 4. Discussion

In the present study, exosomes from AD-MSCs were successfully isolated, and the therapeutic potential for repairing skeletal muscle defects using MSC-derived exosomes was revealed.

This study used human AD-MSCs because of their advantages compared with MSCs from other origins. The adipose tissue can be harvested more easily and safely than the bone marrow or birth-associated materials. Restriction is less on the amount of adipose tissue. Contrary to the bone marrow, in which differentiation potential, number, and maximum life span decrease significantly with age [24,25], the differentiation capacity of AD-MSCs has been reported to be maintained with age [26]. The low immunogenicity and modulatory effects of AD-MSCs allow for their clinical applications in allogeneic transplantation and in therapies for treating immune-resistant disorders [27,28]. AD-MSCs have shown angiogenic and antiapoptotic capabilities, facilitating tissue regeneration, mediated by the secretion of cytokines including growth factor, IGF-1, VEGF, HGF, granulocyte/macrophage colony-stimulating factor, and cell-derived stromal factor 1-alpha [14,15,16,29]. With these features, AD-MSCs have been applied for the regeneration of various tissues.

Regarding skeletal muscle regeneration, AD-MSCs are considered a potential candidate for muscle regenerative therapies. Differentiation toward the myogenic lineage of AD-MSCs has been reported [30,31,32]. In their comparative study, Bayati et al. showed similar in vitro myogenic potential between bone marrow- and adipose-derived stem cells and concluded that AD-MSCs might be useful in skeletal muscle regeneration because of their easy isolation and capacity for rapid expansion in just a short period [33]. The muscle regenerative effect of AD-MSCs was also reported using an in vivo muscle injury model. Gorecka et al. showed that transplantation of AD-MSCs significantly improved functional muscle tissue regeneration in an injured muscle [17]. Furthermore, Zimowska et al. reported that cytokine-pretreated AD-MSCs had a beneficial effect on skeletal muscle regeneration [34].

However, for successful muscular regenerative therapies, it is necessary that transplanted AD-MSCs not only engraft in muscles but also differentiate efficiently, resulting in a positive functional output. MSC therapeutic effects might be due to the secretion of bioactive factors in the extracellular vesicles [35]. Gorecka et al. suggested that AD-MSC facilitates muscle regeneration by secreting factors involved in transient acceleration of skeletal muscle repair [34]. In this context, stem cell-derived exosomes have recently been introduced to regenerative medicine. Stem cell-derived exosomes have several advantages over cell-based therapy: (1) no host immune response because of low immunogenicity, (2) no tumor formation because of their inability to self-replicate, (3) no risk of infection transmission, (4) a low amount of isolated cells required compared with cell-based therapy, (5) ease of storage and increased resistance to freezing and thawing than stem cells because of their lipidic structure, and 6) the presence of specific products (i.e., nucleic acids and proteins) that can be easily obtained [36].

Although the underlying molecular mechanism remains unclear, some preclinical studies have confirmed that MSC-derived exosomes show promise in the field of regenerative medicine through stemness maintenance, apoptosis inhibition, and induction of regenerative phenotypes.

MSC-derived exosomes encapsulate stem-associated transcription factor mRNAs and miRNAs, including Nanog, Oct4, HoxB4, and Rex-1, which play crucial roles in maintaining stem-cell characteristics [37]. Khan et al. showed that MSC-derived exosomes could protect cardiac progenitor cells and promote cell proliferation in a murine model of myocardial infarction by delivering miR-294, which eventually led to improved myocardial function [38]. In another study, McBride et al. showed that Wnt3a, transported by MSC-derived exosomes, binds to LRP6 receptors to promote dermal fibroblast proliferation in vitro [39]. Similarly, MSC-derived exosomes can deliver CD73 proteins to activate AKT and ERK signals and to improve chondrocyte vitality in vitro [40].

Moreover, MSC-derived exosomes can protect against cell apoptosis induced by several factors and reduce tissue damage. Yao et al. revealed that MSC-derived exosomes transported mitochondria-located antioxidant enzymes and manganese superoxide dismutase, which inhibited hepatocyte apoptosis induced by oxidative stress and protected against hepatic IRI in rats [41]. EVs produced from amniotic fluid stem cells could capture excess VEGF through VEGF receptor-1 on their surfaces to protect against VEGF-induced glomerular endothelial cell apoptosis in mice [42].

MSC-derived exosomes have regenerative phenotypes, including self-repair and tissue regeneration, which accelerate the regeneration of injured tissue and maintain tissue homeostasis. This phenotype works in three phases: inflammation, proliferation, and remodeling [43].

During the inflammation phase, MSC-derived exosomes switch macrophage signaling to differentiate toward M2 phenotypes (anti-inflammatory) [44], which accelerate wound healing and inhibit excessive scar formation [45]. For example, Li et al. showed that, during intense burn-induced inflammation, miRNA-181c-carrying exosomes reduced severe inflammation by downregulating the Toll-like receptor 4 signaling pathway [46]. During the proliferation phase, the steps for neo-angiogenesis, collagen deposition, and granulation tissue formation occur [47]. Some studies have targeted this phase as responsible for tissue regeneration. For example, Li et al. showed that endothelial progenitor cell-derived exosomes increased angiogenesis signaling molecules including VEGF-A, VEGF receptor-2, and fibroblast growth factor-1 [48]. Moreover, Hu et al. revealed that, when MSC-derived exosomes are incorporated into fibroblasts, they stimulate cell migration and proliferation by increasing the gene expression of N-cadherin, cyclin-1, PCNA, and collagens I and III [49]. During the remodeling phase, Zhang et al. showed that MSC-derived exosomes accelerate cartilage and subchondral bone regeneration by promoting the formation of neo-tissue and extracellular matrix (ECM) including type II collagen and sulfated glycosaminoglycan [50]. Particularly, miR-125b and miR-320 have also shown therapeutic effects by modulating ECM formation [51,52,53,54,55,56,57,58,59,60,61,62,63].

Skeletal muscles injured by laceration secrete exosomes enriched in myogenic growth factors that subsequently stimulate differentiation of AD-MSCs toward a myogenic lineage to assist in muscle regeneration [54]. Regarding skeletal muscle regeneration by MSC-derived exosomes, Phinney et al. reported that the bone marrow MSCs secrete vesicles that stimulate MYOD and myogenin, which facilitate myofiber regeneration in target cells [55]. Moreover, MSC-derived exosomes have been known to attenuate fibrosis, improve capillary density, and accelerate the regeneration of injured muscles [56]. This is largely accomplished via the cellular transfer of vesicles that contain growth factors or cytokines (IGF-1, HGF, TGF-B1, FGF-2, VEGF, PDGF, and IL-6), in addition to exosome-enriched miR-1, miR-133, miR-206 (containing EXO motifs GGAC, CCCU, and CCCG, respectively), miR-125b, miR-494, and miR-601 that promote a variety of pro-regenerative cellular processes [57,58,59,60]. Similarly, Guescini et al. reported that oxidatively injured myotubes promote satellite cell proliferation through vesicle-mediated repression of myogenin expression in target satellite cells, resulting in faster wound closure in an in vitro wound assay [61].

As discussed above, tissue regeneration through exosomes has been actively studied for skin, cartilage, and skeletal muscle injuries. However, regarding the frequency of study, research regarding muscle regeneration by exosomes is very scarce. Moreover, although the interaction with the surrounding environment plays an important role because of the nature of exosome therapy [62,63], no remarkable studies were found for muscle regeneration using exosomes from AD-MSCs that consider the surrounding environment. Therefore, observing the regeneration of the muscle with an in vivo model whose surroundings are similar to real-world settings rather than in vitro experiments is important. We believe that our rat model study will be an important step for exosome utilization for muscle regeneration, with the end goal of resolving sarcopenia.

Extracellular vesicles of an average diameter of 118 nm were found containing CD9, CD81, and CD63 in this study. These findings are consistent with previous reports about exosomes in the size of vesicles and the presence of proteins localized in the membrane of exosomes [64]. As per the hypothesis, the therapeutic potential for the repair of skeletal muscle defects using MSC-derived exosomes was revealed. Through MSC-derived exosome treatment at the cellular level, increased satellite cell viability was observed, and the activity of the markers for myogenic differentiation was significantly increased. Furthermore, improved muscle regeneration was determined in the mouse muscle defect models. Muscle regeneration, re-established muscle integrity, and reduced inflammatory cells were observed in muscle treated with exosomes. The negative control (treated with gel only) also showed regeneration; however, necrosis and inflammatory cell infiltration were still found in the negative control group. Our results show that exosome treatment accelerates muscle regeneration.

It is noteworthy that muscle loss caused by aging or chronic/genetic diseases attracts more interest than traumatic muscle injury. In chronic muscle disorders, such as acute muscle injury, the infiltration of neutrophils and M1 macrophages is prominent [65]. Additionally, M2 macrophages are also found at the early phase of inflammation and inhibit the production of NO and lysis of muscle cells by M1 macrophages [66]. M2 macrophages also activate eosinophils that promote fibrosis by inducing cytokines [67]. Therefore, the late stage of muscle regeneration in chronic muscle disorders features pathological fibrosis [68]. In these conditions, activation of satellite cells in the whole body is necessary for facilitating protein synthesis and muscle regeneration. Alternative to the pharmacologic approach, which failed to show functional improvement, systemic administration of AD-MSC- or AD-MSC-derived exosomes may be a potential treatment for these conditions.

This study had several limitations worth mentioning. First, unlike other kinds of exosomes that show therapeutic effects, the mechanism of AD-MSC-derived exosomes was not fully understood in this study. Although some miRNAs were reported to be effective and have proven mechanisms in treating the skin, cartilage, and other tissues, their effect on muscles still lacks evidence regarding miRNA or another underlying signaling pathway. This study was difficult since the cargo of MSC-derived exosomes was diverse, and the basic function of exosomes is environmental to intercellular communication, which means that the interaction of the participants and the signaling pathways are numerous; additionally, analyzing the underlying regenerative mechanism separately was difficult. This is why exosome experiments should have consequential interpretations [69]. Second, only negative controls using gel alone and AD-MSC-derived exosomes were compared in this study. Including bone marrow-derived MSCs or satellite cells as a control is desirable for study design. However, the aim of the present study, which was to investigate the muscle-regenerating effect of AD-MSC-derived exosomes, could be identified using the present study design. Third, the modes were injured via punch biopsies, which is a local injury and not systematic. From this model, finding the effects on the whole-body sarcopenia model that requires systematic treatment of exosomes is challenging. Therefore, systemic administration was not attempted in this study. However, because the effectiveness of local treatment has been sufficiently verified, the study can be advanced to develop exosomes that can be maintained through the circulatory system.

## 5. Conclusions

In conclusion, our results suggest that human AD-MSC-derived exosomes may be useful for repairing damaged muscles by promoting the regenerative potential of satellite cells. Further studies are needed to clarify their treatment potential beyond natural regeneration capacity and to investigate whether they can be used for muscle regeneration in clinical settings.

## Figures and Tables

**Table 1:** List of antibodies used in this study.

Antibody	Company	Species	Dilution
CD9	Invitrogen	Human	1:1000
CD63	Santa Cruz	Human, rat, mouse	1:1000
CD81	Invitrogen	Human, rat	1:1000
MYOG	Santa Cruz	Human, rat, mouse	1:300
MYOD	Santa Cruz	Human, rat, mouse	1:300

**Table 2:** qRT-PCR target gene and used primer sequence.

Gene		Primer Sequence (5′→3′)
*M* *YOG*	Forward	GGGGAAAACTACCTGCCTGTC
	Reverse	AGGCGCTCGATGTACTGGAT
*MYOD*	Forward	CGCCATCCGCTATATCGAGG
	Reverse	CTGTAGTCCATCATGCCGTCG
*PAX7*	Forward	ACCCCTGCCTAACCACATC
	Reverse	GCGGCAAAGAATCTTGGAGAC
*GAPDH*	Forward	ACAACTTTGGTATCGTGGAAGG
	Reverse	GCCATCACGCCACAGTTTC

MYOG: myogenin (myogenic factor 4); MYOD: myogenic determination 1; PAX7: paired box 7.

**Table 3:** Experimental scheme for three NOD-SCID groups.

Group	Surgery	Scaffold	Exosome	Animal (n)
Healthy control	Mock surgery without muscle defect	N/A	-	4
Gel alone	Muscle defect	Fibrin	-	6
Gel + exosome	Muscle defect	Fibrin	+2 mg/mL	6

## Data Availability

The data presented in this study are available on request from the corresponding author.
